# Narrative Medicine: A Digital Diary in the Management of Patients with Bone and Soft Tissue Sarcoma—A Multidisciplinary Pilot Study

**DOI:** 10.3390/jcm12237218

**Published:** 2023-11-21

**Authors:** Maria Cecilia Cercato, Concetta Elisa Onesti, Sabrina Vari, Gabriella Maggi, Wioletta Faltyn, Serena Ceddia, Irene Terrenato, Virginia Scarinci, Francesca Servoli, Cristina Cenci, Roberto Biagini, Virginia Ferraresi

**Affiliations:** 1 Epidemiology and Tumor Registry Unit, IRCCS Regina Elena National Cancer Institute, 00144 Rome, Italy; cecilia.cercato@ifo.it; 2 Sarcomas and Rare Tumors Departmental Unit, IRCCS Regina Elena National Cancer Institute, 00144 Rome, Italy; elisa.onesti@ifo.it (C.E.O.); virginia.ferraresi@ifo.it (V.F.); 3 Psychology Unit, IRCCS Regina Elena National Cancer Institute, 00144 Rome, Italy; gabriella.maggi@ifo.it; 4 Orthopaedic Oncology Unit, IRCCS Regina Elena National Cancer Institute, 00144 Rome, Italy; wioletta.faltyn@ifo.it (W.F.); roberto.biagini@ifo.it (R.B.); 5 Biostatistics and Bioinformatics Unit–Scientific Direction, IRCCS Regina Elena National Cancer Institute, 00144 Rome, Italy; irene.terrenato@ifo.it; 6 Digital Library ‘R.Maceratini’, IRCCS Regina Elena National Cancer Institute, 00144 Rome, Italy; virginia.scarinci@ifo.it (V.S.); francesca.servoli@ifo.it (F.S.); 7 Digital Narrative Medicine (DNM s.r.l.), 00161 Rome, Italy

**Keywords:** narrative medicine, bone sarcoma, soft tissue sarcoma, digital diary

## Abstract

Although patient narratives have been increasingly introduced in various fields of medicine, a standard method in clinical practice is still lacking. The objectives of this pilot study were to evaluate the feasibility and usefulness of a digital narrative diary integrated into the care pathway of patients with bone sarcoma and limb soft tissue sarcoma both from the patients’ and the healthcare professionals’ (HCPs) perspectives. A digital platform, DNMLAB, was designed to obtain guided narratives from patients during their pathway of care in compliance with confidentiality and data protection laws. The diary was used for patients, often young, facing a rare and impactful disease that is difficult to manage and with few opportunities to share experiences. The multidisciplinary team shared the narratives and integrated them into the patient’s treatment pathway. Narrative prompts were adequate for the care pathway. Patients correctly considered the diary as a shared area to think about their condition, and HCPs considered it “a shared area growing at each meeting with the patient”. The main advantages reported by patients were increased awareness, the opportunity to express their opinion on cures and important personal needs and the perception of better taking charge (score ≥ 4.6). The main advantages of HCP were improved communication, therapeutic alliance, and deeper knowledge of patients. This study confirmed the authors’ previous experiences, showing that a digital narrative process is feasible and useful for oncology clinical practice according to patients and HCPs.

## 1. Introduction

Interest in patients’ experience of illness emerged around the 1970s [1]. Early studies focused on exploring the subjectivity and meaning patients gave to illness through metaphors, cognitive representation, and imagery [1]. The underlying assumptions of these studies were based on Kleinman’s classification of the concept of pathology into three different areas: disease, intended as a biomedical description of a condition; illness, concerning the patient’s personal and emotional perception; and sickness, focusing on social and cultural aspects [2]. The concept of narrative medicine was subsequently systematized by Charon et al. and introduced into the clinical practice. The aim was to overcome the traditional view of the psychiatric and psychological sciences and leave space for a shared approach by all the actors involved in the care pathway (patients, medical doctors, nurses, psychologists, etc.) [3]. This translates into listening to the patient’s daily needs and better personalizing care by bridging the gap between evidence-based medicine, a purely scientific approach, and the patient’s subjective and emotional views, needs and expectations [4,5,6].

Patient narratives have been increasingly introduced in various fields of medicine. However, only in 2015 were recommendations for the use of narrative medicine in rare and chronic degenerative diseases published by the Italian Istituto Superiore di Sanità [7]. A systematic literature review published in 2016 showed that the use of narrative medicine was based on different methods, such as storytelling, autobiographical telephone interviews, drawing, and theater acting. However, no common specific research methodology was used to assess the impact of narrative on medical practice and patients’ lives [5]. Although narrative medicine application was not methodologically uniform, it benefited the patient and improved the doctor–patient relationship.

To test valid tools for introducing narrative medicine into oncology clinical practice, several pilot studies have been carried out since 2015 at the Istituto di Ricovero e Cura a Carattere Scientifico (IRCCS) “Regina Elena” National Cancer Institute in Rome [8,9,10,11]. In 2017, a digital narrative diary implemented on an online platform (DNMLAB) was introduced and tested in different oncology settings [12]. The diary was designed to collect theme-oriented patients’ narratives during the care path and help healthcare professionals (HCPs) integrate the patient’s perspective and needs with clinical data to personalize the cure [12]. In the first study, we enrolled patients affected in the breast or colon cancer receiving chemotherapy and patients with solid tumors receiving radiotherapy [9]. Overall, 46 patients were enrolled, 31 using the DNMLAB diary. According to the patient and HCP evaluation, the digital diary was considered easy to use, suitable for the context, and useful for communication, care relationship, therapeutic alliance, awareness, coping skills, and information gathering. In particular, the medical staff pointed out that through the diary, they had become aware of personal elements of the patients that they had never mentioned during the previous outpatient consultations. The results provided the basis for exploring the role of the digital diary in other oncology settings of our institute.

The “Regina Elena” National Cancer Institute is a member of EURACAN (EUropean network for RAre adult solid CANcer) and a referral center for sarcomas. Soft tissue sarcomas (STSs) and bone sarcomas (BSs) are rare tumors, accounting for less than 1% of total malignancies, with an incidence in Europe of 4–5/100,000 inhabitants/year for STSs and 0.8–0.9/100,000 inhabitants/year for BSs [13,14,15]. They can appear at any site in the body and often affect the young population with a first peak in childhood and a second peak after the age of 60 [14,15]. The type of pathology and treatments, often including demolition surgery, greatly impact the patient’s autonomy and physical appearance. This, along with young age and long duration of highly toxic chemotherapy regimens, often leads to emotional repercussions, requiring special attention from the caregiving perspective. A multidisciplinary approach and methods closer to younger patients’ mindset, such as digital story sharing, can help establish a relationship of trust with the healthcare team and bring greater awareness of the disease and acceptance of the new condition.

The objectives of this pilot study (*“AMENAS: Application of narrative medicine in the treatment of sarcoma patients”*) are to evaluate the feasibility and the usefulness of the digital narrative diary integrated into the care pathway of patients with BS and limb STS both from the patients’ and the HCPs’ perspectives.

In the preliminary analysis, patients and HCPs rated the digital diary tool as supportive and useful; it improved patients’ awareness and self-empowerment as well as the relationship between patients and clinicians and among the multidisciplinary care team members [10]. The full results are reported here.

## 2. Materials and Methods

### 2.1. Study Design and Ethical Considerations

This prospective descriptive mixed, qualitative–quantitative study was conducted in a real-life setting at the IRCCS “Regina Elena” National Cancer Institute in Rome to evaluate the feasibility and usefulness of introducing a digital narrative diary into clinical practice. The study was approved IFO-Bietti Foundation IRCCS Central Ethic Committee of the Lazio Region (Registro Sperimentazioni n. 1213/19; 04-16-19). Patients gave their written informed consent to participate in the study, to use the DNMLAB digital narrative diary, and to the confidential data-handling procedures for research and assistance according to the Italian Law 193/2006 on Privacy and Safeguarding of Sensitive Data and the General Data Protection Regulation of the European Union 2016/279 (Personal data code protection, Regulation (EU) 2016/679) [16,17]. This project was performed according to the principles of the Declaration of Helsinki. Patients’ priorities, experiences, and preferences were constantly considered while collecting digital narrative outcomes. Patients were not directly involved in the study’s design but were informed of the research outcomes through personal communications with HCPs.

### 2.2. Patient Population and Healthcare Professional Team

Patients with STSs or BSs, irrespective of the stage of disease, aged ≥18 years, undergoing chemotherapy (±surgery), as out- or in-patients at the Sarcoma and Rare Tumors Departmental Unit of our Institute were included in the study. Other inclusion criteria were the ability to write in Italian, the availability of an e-mail address, and the ability to access the DNMLAB platform via a suitable electronic device. Exclusion criteria were poor compliance, the presence of psychiatric disorders, and the presence of severe cognitive deficits.

The HCP team involved consisted of three oncologists, a psychologist, and a nurse case manager who were trained in using the DNMLAB digital platform before the start of the study. The team involved in the development of the study design, the analysis of the digital diaries, and the interpretation of the data were also composed of other professionals with expertise in the application of narrative medicine in research projects and qualitative research (an oncologist/epidemiologist, an anthropologist, a literature graduate, and librarians), and one technical referee for the use of the digital tool.

### 2.3. Intervention

HCPs invited the eligible patients, providing them with e-mail access to the diary, monitoring them by reading their stories, and then sharing and using these during the scheduled visits or hospitalization to personalize their treatment. Patient access to the digital diary was granted only upon invitation by an HCP, and the system met all the criteria of healthcare data protection required by the Privacy Authority and European regulation. The patients entered the platform via a computer or smartphone and digitally signed the Consent for Participation in the study. The DNMLAB gave the patient access to a protected personal area called a ‘diary’, where they could write their narrative, which was guided by a set of narrative prompts developed by the team. The prompts were conceived according to each patient’s path of treatment, depending on the phase of the care in which the patient was enrolled, with a list of six prompts for patients undergoing chemotherapy, a list of four prompts for patients undergoing surgery, and two lists of seven and three prompts, respectively, for patients in follow-up. The platform sent the prompts to patients twice a week, and the patient could choose to respond or skip them. In case of a non-response, the patient did not receive the next prompt. The response to each prompt had a character limit of 2000–3000. The narrative could be supplemented by free text from the patient at any time.

Each HCP examined the text and discussed it with the team and the patient during the scheduled visits.

The study duration was 15 months. The collection of narratives took place over a 12-month period: the diaries’ cut-off date was considered 1 December 2022.

### 2.4. Endpoints

The main endpoint of this study was the feasibility of the DNMLAB platform. The measure of feasibility was evaluated by HCPs in terms of ease of use and management time spent and by the patients regarding accessibility, ease of use, and satisfaction.

Secondary endpoints were usefulness and value for clinical assistance purposes, namely: opinions of HCPs about improvements in communication, care relationship, interdisciplinary relationship, therapeutic alliance, and collection of information that would otherwise be unattainable; patients’ opinions regarding the ease of producing a story, effectiveness in terms of being better listened to and understood, initiation/reinforcement of a therapeutic alliance (between patient and caregiver and among team members), and increased awareness and ability to cope with their disease. HCPs and patients rated the items through a self-assessment questionnaire, including open and closed questions (Table 1 and Table 2). Open questions included comments and suggestions. According to the study aims, HCPs carefully read, interpretated and integrated their patients’ narratives into the clinical practice by blending them with clinical data, shaping individualized care paths for each patient.

Answers to closed questions were scored on a 5-point Likert scale from 1 = complete disagreement to 5 = complete agreement [18]. The questionnaire was administered to patients at the end of the collection of the stories; HCP evaluation was required twice: at 6 months and the end of the study. Patients who had not written in the diary answered a questionnaire to assess non-participation, which was structured into multiple-choice questions and an open-ended question.

### 2.5. Statistical Analysis

In line with a pilot exploratory study, the sample size was defined to assess the objectives using descriptive analysis. Continuous variables were presented as mean and standard deviation (SD) or median and range, while categorical variables were reported as frequencies and percentage values. The Mann–Whitney nonparametric test was applied to compare the two healthcare professional evaluations. After being invited by e-mail, patients were considered participants in the study if they responded to at least one narrative prompt.

The study aim did not include a qualitative analysis of the texts to detect the experiences and needs of people with sarcoma. Two HCPs (CEO, SV) and two additional researchers on the team (the oncologist/epidemiologist (MCC) and the anthropologist (CC)) read all the texts (narratives, open-ended questions and comments) and used them in order to interpretate and deepen the questionnaires items assessment. Texts were analyzed manually or using a word cloud generator available in DNMLAB. The results were presented and discussed among the entire research team. In this study, we used the SQUIRE reporting guidelines [19].

## 3. Results

From January 2021 to December 2022, a total of 30 patients were enrolled in the study, 21 (70%) of whom completed the registration on the DNMLAB platform and 17 (56.7%) of whom participated in the digital diary (at least one reply to prompts). The mean age of participating patients at the time of inclusion in the study was 41 years (standard deviation (SD) 8.2); six (35.3%) were males and 11 (64.7%) females, nine (52.9%) were affected by STSs and eight (47.1%) were affected by BSs. Most patients presented a localized stage of disease (12, 70.6%). The full characteristics of adherent and non-adherent patients are summarized in Table 3.

Overall, at the cut-off date on 1 December 2022, HCPs had assigned 35 lists with 120 prompts. Patients completed 14 (40%) lists, visualized 109 out of 120 (91%) received prompts, and answered 98 of them (82%). Two patients wrote without a prompt, with 34 free texts, and uploaded eight personal or family pictures in the diary.

In addition, 16 of 17 adherent patients completed the evaluation survey, while one died before questionnaire administration. Patients gave a score on a 5-point Likert scale, where the higher score corresponded to the highest degree of agreement: for all the items of feasibility and usefulness the mean score ranged from 4.4 to 4.8 (Table 1).

Five HCPs (three oncologists, one psychologist, and one case manager nurse) answered the evaluation survey in the middle and at the end of the study. At first evaluation, a mean score > 4 for all the items of feasibility and usefulness was observed except for time management to propose/evaluate the diary use (mean score 3.8; SD, 0.45) and for visit length optimization (mean score 3.2; SD, 0.84). At the end of the study, we observed an improvement in these two items but a worsening in the score for team relationships, although the differences were not statistically significant (Table 2). With regard to time management, HCPs reported that they used only a few minutes on average to read patients’ narratives immediately before the scheduled visit; in addition, this allowed them to make the most of the duration of the visit by placing the focus on emerging aspects promptly.

Although the non-adherent patients did not use the diary, they gave a score of 4.7 (SD, 0.63) for the potential improvement in personalization of care and quality of life using the digital narrative diary. Most of them stated that they did not participate because of technical problems (38.5%) or because they preferred to speak verbally during the visits (38.5%). Reasons for lack of participation, intentions regarding the use of the diary, and habitual use of the internet are summarized in Table 4.

### 3.1. Qualitative Results

All narratives, comments, and texts from the open-ended questions were considered in order to interpretate and deepen the questionnaires items assessment. Word clouds for each patient were also generated and interpreted by using them as a subsidiary tool. Emerging data were analyzed within the researcher team and grouped into two main themes: (a) the role of digital diary as a tool for self-expression and (b) the utility of the digital diary for communication and care relationship.

### 3.2. The Role of Digital Diary as a Tool for Self-Expression

Patients strongly agreed with the questionnaire statements that the diary offers the opportunity to provide “important personal information”, “information that one did not want/could not communicate verbally” and to “express one’s point of view”.

The digital setting, diary format, and narrative prompts seemed to have enabled each person to express his or her point of view to the fullest. In fact, the guided, but at the same time, free format allowed each person to choose how to use it and how to tell his or her story. Very different conversational strategies were found. Here are some examples.

A 50-year-old female patient thought the diary was a personal area rather than something to be shared with the treating personnel: *“Dear diary, I have confided in you my thoughts for a short time; I realized that you could help me”.* She often used free narrative (31 times), reflecting on her relationship with her daughter and including photos of herself and her family and metaphoric images. The diary shaped a new “start” by relating the resources available to her to cope with the disease.

Conversely, another female patient answered very synthetically to all prompts without free texts or images. This patient chose for her writings the feature of a testimony addressed not only to HCPs but also to all those who lived her experience. *“I feel like suggesting that anyone in my condition must accept failures; there will be failures, but they are not the focus. Meanwhile, let’s enjoy anyone who loves you and any simple thing that makes you happy”.*

Sometimes, the patients used the diary to discuss their needs directly with the treating team. A male patient wrote about his displeasure and criticized the assistance organization.

Although with different styles and aims, all the patients understood correctly that the diary was intended as a tool for sharing thoughts about their condition. No one used it inappropriately (as a chat or a messaging service).

### 3.3. The Utility of the Digital Diary for Communication and Care Relationship

HCPs rated a high score to questionnaire items concerning the improvement in communication and care relationship achieved by introducing the diary in clinical practice. The text enabled the treating team to detect some features of the illness experience that were useful in setting the communication on the correct tone, which is pivotal in the therapeutic relationship and, consequently, in treatment adherence. Perception of time is a meaningful example: time of disbelief, “*I absolutely could not link that nasty word (cancer, editor’s note) with myself*”; time of previous life, “…*that lasts as a remembrance, now*”; the suspended time of waiting (i.e., of a diagnostic test), “*time stopped(at disease development, editor’s note)*”; changes in everyday life rhythm, “*my body needs time*”, “*my body sets me in slow motion*”; future, “*in the short course*”, “*living from day to day*”, “*my tomorrow is getting out of the hospital… I have no idea where to start and, mainly when to start*”.

It was important for the treating team to be able to integrate the clinical perception of time, related to the course of the disease, with each patient’s subjective time, which was associated with alternating intervals of acceptance and denial of the disease, experiences of liminality (suspended/stopped time) and the need for a normal daily life [20].

The narrative often made the personal way of coping with disease and treatment explicit, showing the individual resources that the treating team should leverage. Patients often used metaphors that seemed important to the communication and the relationship between caregiver and patient. The treatment course was often narrated as a journey to exorcise the impact of the disease, including in social and work relationships: “*My cure includes a hospitalization at each cycle. It is a journey for me… I always tell my colleagues that I am leaving to communicate my hospitalization days, and I always choose a new destination*…”; “…*enjoying the complimentary cruise*”; “*the adventure*”; “*climbing to the mountaintop; you do not have to look all the way up each time, because it seems endless, but you have to get to each turn of the path and keep going, gazing up at the top from time to time*”.

Other meaningful metaphors concerned ambivalence toward the cure that was described as a “*poison*” but also as an “*ally*”; the hospital, which was described as a “*nightmare*”; patients as “*walking deceased*” and the “*burden*” of others’ stories and suffering carried with you but also as “*comrades*” who “*remain in your heart*”; disbelief and upset at the diagnosis, receiving which was described as “*as in a dream*”, “a *bolt from the blue*”, “*my perfect storm*”; to cope with cancer as a “*monster*”, “*intruder*”, “*fight*”, “*battle*”, “*failure*” but also a “*gift from life*”.

Finally, a careful assessment of illness narratives was a powerful tool to detect perceptions of treatment quality and to shape the organization and content of the assistance mode based on patient expectations/needs.

HCPs’ comments agreed on the usefulness of the tool in improving the treatment relationship regardless of the professional role: “*The use of this digital diary allowed me to learn about some aspects of patients’ daily life that I was unaware of. This has led me to be more emphatic towards them; however, the emotional impact of this diary on healthcare professionals deserves to be carefully evaluated*” (oncologist, female, 36 years old). “*The diary was useful in understanding some emotional and personal problems beyond the disease under treatment. It was possible to develop an empathy that improved the physician-patient relationship…”* (oncologist, female, 40 years old). “*Understanding better, the main point is that a more emphatic relationship could be initiated with that patient*” (case manager nurse, female, 50 years old). “*When you get insight into the patient’s narrative, this is the added value of the tool; you can develop a higher sensitivity, which improves your ability to take care and promotes emotional understanding*” (psychologist, female, 57 years old).

Additionally, the diary practically helped to include narrative elements into the care pathway that could be tailored according to the needs and expectations of each patient. “*It helped me in the communication with the patient, in the management of their care pathway, resulting in an improved adherence to treatments*” (case manager nurse, female, 50 years old). “*It enabled me to know the patient’s priorities, which are often unspoken and do not always overlap with the aims of the care pathway or with the physician’s objectives, even for life-threatening diseases such as malignant tumors; identify possible caregivers or pivotal kins to set an effective alliance, useful for the management of the care pathway; understand better the inner world of the patient to be able to shape communication modalities of bad news (possible recurrences, therapy changes due to treatment failure)* …” (oncologist, female, 55 years old). “*I could appreciate this valuable tool in my clinical practice, which enables us to read what they wrote in the diary before each meeting so that by sharing their experiences, the relationship is emphatic. The use of selected metaphors as a background of the notation made the communicative relationship not only words exchange but an intimate relationship that transmits, by images, listening, understanding, participation, sharing*” (psychologist, female, 57 years old).

In the practice and organization, one issue is shown: “*The possibility of identifying the weak link of multidisciplinary interventions in tumors such as sarcomas, which are usually followed simultaneously by specialists from different departments of our center (i.e., for chemo-radiotherapy, post-surgery complications, rehabilitation during systemic therapies…)*” (oncologist, female, 55 years old).

Eventually, the words of HCPs showed an important feature of narrative medicine: the possibility of supporting the treating personnel with a personal benefit during the clinical activity: “*A personal benefit of the tool was, sometimes, to support the burden of work, by the psychological point of view*” (case manager nurse, female, 50 years old).

## 4. Discussion

Over the past few decades, narrative medicine has progressively attracted interest. However, a standard method in clinical practice is still lacking [5]. Several narrative research studies on illness narratives and parallel charts (personal notebooks in which clinicians can write reflections and feelings) have provided insights for clinical practice and healthcare service organizations [21,22].

Given the increasing use of the Internet and social media to share content, especially among young people, storytelling on an online platform seems simple and timely. An innovative and nonprofit start-up (DNM S.r.l.) created a digital platform, DNMLAB, which was designed to obtain guided narratives from patients during their pathway of care in compliance with confidentiality and data protection laws [12]. At the “Regina Elena” National Cancer Institute in Rome, several pilot studies using the DNMLAB digital narrative diary have been carried out since 2017 to improve the oncology clinical practice for cancer patients in different care settings [8,9,10,11]. Furthermore, data concerning the beneficial use of the digital platform DNMLAB in several clinical conditions, such as heart failure, cardiovascular disease, and epilepsy, were also reported [23,24]. Overall, the results support integrating patients’ narratives with clinical data and encourage further research.

This study has some innovative features compared to previous ones: (a) a rare disease with very difficult management; (b) young patients facing an impacting disease (severe prognosis, aggressive and sometimes mutilating therapies, less opportunity to share their experience in comparison with more common tumors); (c) HCPs were involved as a multidisciplinary team and not personally (oncologist, psychologist, nurse). The team shared the narratives and integrated them into the patient’s treatment pathway; each used them according to their professional role, in a triangle relationship of patient–professional and professional–professional, with an additional benefit in building therapeutic alliances.

This study confirmed the authors’ previous experiences, showing that a digital narrative process is feasible and useful for oncology clinical practice according to patients’ and HCPs’ perspectives. Narrative prompts were adequate to the care pathway and did not limit the free expression of patients, who also produced free narratives, selfies, family photos, and metaphoric images. Although the patients used different narration and communication registers, the diary was correctly considered as a shared area to think about their condition, and the HCPs perceived it as “a shared area growing at each meeting with the patient”. The main advantages reported by patients were increased awareness, the opportunity to express their opinion on cures and important personal information, and the perception of better taking charge (score ≥ 4.6). HCPs reported the following advantages: better communication, deeper knowledge of the patient, and increased therapeutic alliance (score ≥ 4.6). HCPs’ scores were mostly higher at the second assessment than at the first one except for the score for usefulness in improving the team’s relationships, which was 4.2 at the first evaluation and 3.6 at the second evaluation. This result may be due to the prolonged COVID-19 pandemic, which made the work harder and required new organizational models. Additionally, it may be due to the current care model that does not include narration as a stable and continuous component of cures [25].

Text analysis and HCPs’ comments showed relevant hints for the use of narrative components in the clinical practice for the management of these patients, who are usually young and need multidisciplinary interventions. The peculiarity of these types of cancer patients, suffering not only from generally aggressive and disabling cancer but at the same time from a rare disease that accentuates the sense of loneliness and difficulty in accessing treatment, contributes to narratives being even more significant. Furthermore, when compared to the narratives collected from patients affected by common cancers (such as breast and colon–rectal cancer), the burden of long periods of hospitalization and the relevance of body impairment emerged. Understanding the “inner world” of the patient may help HCPs adopt tailored communication specifically to convey bad news. Metaphors were often used in narratives and helped HCPs know how the individual coped with the disease. The metaphor allowed the patient to communicate better, reduce emotion in observing themselves, and increase their coping capacity toward the disease, particularly cancer [26,27,28]. Fergus et al. wrote, “In women’s narratives about their experiences of cancer…, metaphors can capture the complex and often contradictory ideas, thoughts and feelings that otherwise would be difficult to describe through the direct expression” [29]. Indeed, metaphor, time and journey themes frequently emerged in that study and ours. The HCP may use the patient’s metaphors to improve communication and make the relationship closer, producing understanding, participation and sharing, resulting in a stronger therapeutic alliance.

Narrative prompts helped express the helping subjects (e.g., the caring kin) and the opponent elements (practical or psychological issues) in living with the disease. Knowing the environment where the patient was coping with the disease contributed to selecting adequate strategies (“building productive alliances functional to the cure pathway”), overcoming barriers, and increasing therapeutic alliance.

Narratives provided information about the perceived quality of cures. Structural, organizational, and relationship concerns may be detected in cure pathways: “possibility of identifying the weak link in the chain of multidisciplinary interventions, in the setting of sarcomas that often involves several specialists”.

Narratives showed that it is important to use the diary in all the phases of cures, including hospitalization; in-person dialogues may not reveal some issues, but written narratives may report important queries to be addressed in the short term. The quality of care and relationships during hospitalization may negatively impact the disease experience and the capacity to use internal resources. Some narratives included sharing experiences with other patients among other elements that may help to cope with the cures: *“Sharing experiences with other people who overcame this problem and who can counsel you”*.

Narratives related to hospitalization periods showed that monitoring and facilitating patient relationships is important to improve cures.

In the future, it might be useful to explore the usefulness of digitally shared narratives within a group that could involve patient association volunteers (who are important in this setting).

Limitations of this study are the reduced number of participant patients and HCPs and the presence of only one excellence center (OECI-certified Comprehensive Cancer Center), so the generalization of results may not be recommended.

On the contrary, a strength of the study is the digital diary, instead of oral narratives, which seems to reduce the unexpressed and facilitate the communication of important issues that are often not shared and that may not be shared in person. Expressing all problems is especially important for patients with a rare disease who have little opportunity to share experiences with other patients.

Although the HCPs used the digital diary to improve communication and relationships, better knowledge of the illness was not used to personalize the cure. This result confirms that it is necessary to produce an environment facilitating the implementation of narrative medicine. More than training is needed to optimize timings and settings; it is necessary to change the clinical approach and redefine the healthcare system in terms of structures, organization, priorities, and shared values in health and disease [5,25].

Indeed, the HCPs’ comments showed that narrative medicine might improve professional skills and provide personal advantages. Further studies are needed to design strategies for HCP well-being and prevent burnout in the oncology setting.

## Figures and Tables

**Table 1:** Evaluation of the digital diary by the patients.

Questionnaire’s Items		Mean Score (SD)
**Feasibility**	Diary easy to use	4.8 (0.45)
	Diary immediacy and comprehensibility	4.7 (0.48)
	Adequacy of one’s computer skills	4.4 (0.81)
	Opportunity to provide important personal information	4.8 (0.54)
	Opportunity to provide information that one did not want/could not communicate verbally	4.5 (0.63)
**Usefulness**	Opportunity to express one’s point of view	4.7 (0.48)
	Perception of being more effectively followed and understood	4.6 (0.63)
	Improved awareness of the disease	4.7 (0.60)
	Improved coping skills	4.4 (0.81)
	Improved care relationship	4.4 (0.89)
	Recommendation to introduce the digital diary in clinical practice	4.8 (0.40)

SD = Standard deviation.

**Table 2:** Evaluation of the digital diary by healthcare professionals.

Questionnaire’s Items		First Evaluation, Mean Score (SD)	Second Evaluation, Mean Score (SD)	*p*-Value *
**Feasibility**	Diary easy to use	4.6 (0.55)	4.8 (0.45)	0.317
	Diary immediacy and comprehensibility	4.6 (0.55)	4.4 (0.55)	0.564
	Time management to propose/evaluate the diary during the visits	3.8 (0.45)	4.8 (0.45)	0.059
	Optimized clinical examination (length)	3.2 (0.84)	4.0 (0.00)	0.102
	Optimized clinical examination (quality)	4.4 (0.55)	4.6 (0.55)	0.564
**Usefulness**	Improved communication	4.8 (0.45)	4.8 (0.45)	1.000
	Improved care relationship	4.4 (0.55)	4.4 (0.55)	1.000
	Deeper knowledge of patient	4.6 (0.55)	4.4 (0.55)	0.317
	Improved therapeutic alliance	4.8 (0.45)	4.8 (0.45)	1.000
	Focus on key points of the care history	4.0 (0.00)	4.0 (0.00)	1.000
	Improved team relationship	4.2 (0.84)	3.6 (0.89)	0.083

SD = Standard deviation. * Mann–Whitney nonparametric test.

**Table 3:** Patients’ characteristics.

	Adherent Patients, n (%)	Non-Adherent Patients, n (%)
Population size	17 (56.7%)	13 (43.3%)
Age (years), mean (SD)	41 (8.2)	45.8 (14.5)
Sex:		
– Males	6 (35.3%)	6 (46.2%)
– Females	11 (64.7%)	7 (53.8%)
Diagnosis:		
– Soft tissue sarcoma	9 (52.9%)	8 (61.5%)
– Bone sarcoma	8 (47.1%)	5 (38.5%)
Disease extension:		
– Localized	12 (70.6%)	10 (76.9%)
– Metastatic	5 (29.4%)	3 (23.1%)
Treatment phase at the time of enrollment:		
– Neoadjuvant	5 (29.4%)	6 (46.2%)
– Adjuvant	4 (23.5%)	2 (15.4%)
– Palliative treatment	4 (23.5%)	3 (23.1%)
– Follow-up	4 (23.5%)	1 (7.7%)
– Surgery	0 (0%)	1 (7.7%)
Medical treatment received:		
– Chemotherapy	12 (70.6%)	7 (53.8%)
– Chemoradiotherapy	5 (29.4%)	6 (46.2%)
Type of chemotherapy@		
– Epirubicin–ifosfamide	9 (52.9%)	6 (46.2%)
– Ifosfamide	4 (23.5%)	0 (0%)
– VAC/VID/EI	2 (11.8%)	2 (15.4%)
– EUROBOSS regimen	1 (5.9%)	1 (7.7%)
– MAP	1 (5.9%)	0 (0%)
– Other	0 (0%)	4 (30.8%)

VAC = vincristine–adriamycin–ciclophosphamide; VID = vincristine–ifosfamide–dactinomycin; EI = etoposide–ifosfamide; EUROBOSS regimen = cisplatin–adriamycin–ifosfamide–methotrexate; MAP = methotrexate–adriamycin–cisplatin.

**Table 4:** Non-adherent patient questionnaire results.

	Patients (n = 13), n (%)
Reason for not using the digital diary:	
*1. I had forgotten the e-mail invitation to participate*	3 (23.1%)
*2. I do not feel comfortable with digital technology*	3 (23.1%)
*3. I found it difficult to use*	1 (7.7%)
*4. I had technical difficulties trying to use it*	5 (38.5%)
*5. I did not feel like talking about myself*	0 (0.0%)
*6. I do not believe it is really useful*	0 (0.0%)
*7. I prefer talking about myself during medical visits*	5 (38.5%)
*8. I did not really understand what it was for*	0 (0.0%)
*9. I was worried that my privacy was not guaranteed*	0 (0.0%)
*10. Other reasons (busy with other commitments)*	3 (23.1%)
Intentions regarding diary use in the near future:	
*1. I will inform my doctor that I do not intend to use it*	0 (0.0%)
*2. I would like to receive a new e-mail invitation to participate*	10 (76.9%)
*3. I will start using it*	9 (69.2%)
*4. I do not think I have enough time for this project*	0 (0.0%)
Habitual Internet use for:	
*1. Search for information*	9 (69.2%)
*2. Access to social network*	9 (69.2%)
*3. E-mail*	9 (69.2%)
*4. Infrequent internet use*	1 (7.7%)

## Data Availability

The data presented in this article were registered on the DNMLAB digital platform and stored in an IFO institutional repository; they will be available upon reasonable request to the corresponding authors.
